# Living labs opened dialogues about antibiotic use in dairy cattle and pig sectors - Insights from a Danish case study based on participatory action research

**DOI:** 10.1186/s13028-025-00816-7

**Published:** 2025-06-17

**Authors:** Mette Vaarst, Merete Studnitz, Mogens Agerbo Krogh, Hanne Kongsted

**Affiliations:** 1 Department of Animal and Veterinary Sciences, Faculty of Technical Sciences, Aarhus University, Blichers Allé 20, Tjele, DK-8830 Denmark; 2 The International Centre for Research in Organic Food Systems (ICROFS), Blichers Allé 20, Tjele, DK-8830 Denmark

**Keywords:** Antimicrobial use, Dairy calves, Multiactor engagement, One-health, Social innovations, Systems transitions, Living labs

## Abstract

**Background:**

Prudent antimicrobial use (AMU) is paramount for future sustainable animal production. Continuous efforts are required to have a low and well-regulated AMU. Living Labs (LLs) are multistakeholder open innovation platforms that have the potential to allow multiple stakeholders to explore common ground, create dialogues and find solutions to jointly identified problems. The purpose of this study was to stimulate multistakeholder dialogues leading to transitions towards a more prudent AMU in the Danish dairy and pig sectors.

**Results:**

The two LLs, which focused on pig farms and dairy cattle farms, worked in parallel for 30 months. Stakeholders in the pig and dairy sectors pointed to systemic structures such as logistic conditions and global market structures as the main drivers of the current AMU. Both LLs expressed conflicting interests and perceptions around the concept of prudent AMU related to animal welfare and today’s production systems. Thus, the LLs in the Danish context became spaces for opening dialogues and negotiating difficult and sensitive issues in addition to being open-innovation platforms. Both LLs expressed scepticism around the connections between AMU in animal farming and the global threat of antimicrobial resistance to humans. However, this scepticism was more dominant for the stakeholders of the pig sector. The feeling of being a global front-runner in prudent AMU, legislation and transparent monitoring of AMU existed in both sectors, but both LLs identified possibilities and room for improvement. The need to reduce AMU was most consistently articulated in the dairy cattle LL, where the stakeholders increasingly focused on calves from dairy farms and the systemic drivers, which made it challenging to reduce AMU. Some actors in the pig LL kept questioning whether a change towards more prudent AMU was needed in Denmark.

**Conclusion:**

The LL approach fostered important dialogues and insights between central stakeholders in the sectors and was thereby helpful in terms of opening and contributing to dialogues about antibiotic usage in dairy cattle and pigs within and across sectors.

**Supplementary Information:**

The online version contains supplementary material available at 10.1186/s13028-025-00816-7.

## Background

The improper use of antimicrobials in many parts of the world contributes to the emergence and spread of antimicrobial resistance (AMR), including the use of antimicrobials for treatment of animals [1–6], although there is still not full consensus on how much AMU in farm animals contributes to AMR in humans [7]. This links it to the concept of One Health, which is defined by the World Health Organization (WHO) as ‘an integrated, unifying approach that aims to sustainably balance and optimize the health of people, animals and ecosystems’ [8]. The AMU used for farmed animals is estimated to constitute between two-thirds [9] and 70% [10] of the global AMU and is still predicted to increase until 2030 [11], although it is generally decreasing in the EU [12]. Perceptions of and guidelines on when and how to use antibiotics in farm animals are consequently under current change and debate among a wide range of actors in human and veterinary medicine as well as the food chain [1, 3].

In Denmark, AMU is low compared to other countries with intensive pig and dairy production. The reported sales of antibiotics to food-producing animals in Denmark in 2021 were 33 mg/Population Corrected Unit (PCU) [13] and an annual use of more than 86 tonnes of active antibiotic compounds for farm animals in Denmark [14]. Countries with comparable pig populations (approximately 10 m. heads) reported sales of antimicrobials for food-producing animals: Poland, 176 mg/PCU; Italy, 174 m/PCU; France, 52 mg/PCU; and the Netherlands, 48 mg/PCU. However, in a Nordic setting, the AMU in Denmark is high [13]. For many years, Danish authorities and industries have worked towards reducing the use of antibiotics, such as imposing restrictions on the use of different types of critical antibiotics before EU legislation did so. Antibiotics are prescribed, but not sold, by veterinarians. Regular visits to herds by a veterinarian to perform herd health advisory services are mandatory due to Danish regulations. These visits can be weekly or monthly, depending on herd size and access to prescription medicine and antimicrobials. All prescribed antibiotics for animals are registered in a national public database, which provides data on antibiotic prescriptions at both farm and species levels [15].

Many studies have focused on farmers’ and veterinarians’ choices regarding antibiotic treatments but have focused less on the drivers and barriers leading to high AMU at a more systemic level, such as regulation, market dynamics and the influence of multiple actors throughout the value chains related to the food, agriculture and pharmaceutical industries [16]. In acknowledgement of the complexity, systemic nature and context dependency of how and when AMU is used, it seems promising to involve local context-relevant actors and approaches to find solutions. This was scientifically evidenced and led to a recommendation that participatory strategic planning approaches that consider contextual factors, a range of actors and impact pathways should be used to design, implement and evaluate plausible and effective interventions [17]. During the last few years, Living Labs (LLs) have been used to innovate and find solutions between stakeholders in the agricultural and food sectors [18–26]. To date, LLs have not been applied with a focus on AMU or to mitigate the risk of AMR [23, 26]. LLs are based on multiactor engagement and are described as user-centred, open-innovation approaches that are responsive to the participants’ specific contexts [24]. LLs have limitations in terms of time and finances and remain dependent on the willingness of stakeholders to participate [27]. On the other hand, LLs open up new perspectives, joint learning and cooperation in new networks. Like much participatory research in agriculture [28], LLs take their point of departure from the participants’ lived experiences. In this project, we used LLs to foster a transition toward a more prudent use of antibiotics because the approach offered a tool for stimulating dialogues and involving stakeholders at different levels of the value chain in cocreating social and technical innovations to facilitate a reduction in AMU at a systemic level. It is described as a “lab” because it combines and integrates processes of cocreation, exploration, experimentation and evaluation of innovative ideas, scenarios, concepts and technologies. An LL’s timeframe can range from months to years [29] but needs to ensure a continuous iterative process [18].

The two LLs in the current study were established as part of an EU project on prudent AMU in farm animals [30]. These studies lasted 30 months and focused on reducing AMU during dairy and pig production.

The aims of this article are (1) to present how the LL processes in this study contributed to engaging stakeholders in dialogues and discussions on AMU within and across the two sectors and (2) to reflect on the potential and value of the LLs to participate in finding solutions towards prudent AMU.

## Methods

### Establishing Living Labs

From April to September 2020, twenty-two semi-structured qualitative interviews were conducted with stakeholders in the Danish dairy and pig sectors. The interviewees were identified through a common process between the first, second and fourth authors, who all had more than two decades of experience with these sectors, and partly through a snowball method, where some interviewees suggested others. The aim of the interviews was to have a broad range of stakeholders represented (please see Additional file 1 here).

Based upon the themes brought forward in the interviews, an initial focus was chosen for the two LLs. For dairy LL, the AMU among calves should be considered. An LL with stakeholders related to the dairy sector as well as the calf sector was identified. For the pig LL, low market prices were identified as crucial barriers to change, and systemic changes were the focus (see Table 1). Approximately 15 central stakeholder representatives per sector were identified and invited to constitute the core groups of two LLs. This number of participants was recommended by the coordination team from the ROADMAP project. They motivated the recommendation of an estimation of feasibility in terms of the time and financial frame of the ROADMAP project to address and reach changes towards common visions on prudent AMU.

**Table 1:** Presentation of the two Danish living labs (LLs), October 2020-May 2023

Sector	The sectors at a glance	Agreed Aim and focus of Living Labs	Participants of LL core group	Main case in this article	Thematic groups (subgroups of the LL core group)
Pig	The Danish pig sector comprises approximately 12.4 million pigs divided into 2400 herds, with the vast majority being indoor, conventional herds. Approximately 1300 herds contain sows, giving birth to approximately 30 million pigs annually, which are transported to herds for fattening until 100–120 kg., or live exported (approx. 16 mill.), typically at 30 kg. Approximately 80–90% of the pork is exported to other countries (Animalsk produktion - Danmarks Statistik).	Aim: To initiate a more prudent antimicrobial use (AMU) by creating dialogues within the sector and beyond. The focus: reduce the average antimicrobial use by optimizing the use in barns. Communicate these practices to consumers and thus ensure sales.	11 participants in the LL core group: Pig farm owners, veterinary companies, farm advisory companies, SEGES (https://segesinnovation.com/about-us/), Danish Agriculture & Food Council, Danish Crown (abattoir), COOP (supermarket chain), and Aarhus University.	“The consumer as central stakeholder”. Inspired by the Pure Pork concept, the LL throughout its course focused on how and if the idea of consumers paying extra for meat produced with less antibiotics could be a way forward. It became more and more clear that consumers expect politicians to handle issues on food safety and are mainly interested in paying for improved animal welfare (e.g. organic) if anything. Thus, systemic barriers for change (breeding, economy, challenged staff situation) came more into focus over time.	Two thematic groups with the following objectives: (a) “The app-group”: to design a digital tool for monitoring, evaluating and communicating about antibiotic treatments in herds. (b) “The roundtable group”: to create a dialogue between multiple stakeholders with diverse backgrounds and opinions on subjects related to antibiotic usage in pig herds and antimicrobial resistance. Products: Published report and contribution at the Danish Pig Conference (https://dcapub.au.dk/djfpublikation/index.asp?action=show&id=1497).
Dairy cattle	The Danish dairy cattle sector consists of a total of 2400 conventional and organic farms. More than 85% of Danish milk is produced on conventional farms, where the average milk production is just above 11.000 kg milk/cow/year. Female calves for replacement are either kept with the herds or in associated heifer hotels. Bull calves are most often sold to slaughter calf herds at approximately 3 weeks of age and slaughtered between 8 and 12 months (rosé veal).	Aim: To develop a common platform of understanding of antimicrobial use and resistance. To develop practice tools for a prudent AMU.	11 LL core group participants were from eight types of organisations: SEGES (https://segesinnovation.com/about-us/), the Veterinary Society, universities (Copenhagen and Aarhus), and private companies (Arla Foods, Himmerlandskød (abattoir), Calvex (company focusing on calf care and production), veterinary practices).	“Calves from dairy farms”. Calves were already included from the start, but the focus became stronger along the way, realising that AMU was high, and during the course of the LL, also increasing. The LL realized that the missing link between dairy and calf herds was the main driver of high antimicrobial use in the dairy cattle (cows and calves) in Denmark. This was followed up during the entire project period and the final product from the LL was a common document forwarded to policy negotiations between sectors planned to take place shortly after the closure of the project.	Three thematic groups with the following objectives linked to the aim of the dairy cattle LL: (a) “The dialogue tools for practice use group”: to create practice tools for dialogues at the practical level: bring up AMU in farmer groups, initiate groups for foreign farm workers, producing small videos of ‘best practice’. (b) “The wider dialogue group”: initiate dialogues with agricultural colleges and create debate in professional environments, e.g., at the Danish Cattle Conference. (c) “The legislation group”: to provide legislation recommendations, where a common recommendation paper for future legislation was developed and submitted to the Veterinary Directorate.

Legend: The table contains brief information about the two sectors, the focus and participants in the two LLs, and headlines illustrating the work done by thematic groups formed by stakeholders in the Living Labs

Both LLs were running from October 2020 until May 2023.

Table 1 provides details on the two sectors and their respective LLs.

### Methodologies used in the LLs, including data collection and analysis

The overall methodological approach in this project was participatory action research (PAR), which builds on participatory iterative processes involving all participants to influence the development of the project [31–33]. The methods used to facilitate the dialogues and processes in and between the LLs are briefly outlined in Table 2.

**Table 2:** Methodologies used throughout the LL process, and their application

Methodology	Which LL	Details on application and perceived outcome
**Facilitation**	Cattle & pig	• Meetings facilitated by participating researchers. At the start, the scene was set by facilitators regarding mutual respect and confidentiality. Dialogue methods (respectful and socratic dialogues) and techniques like break-out-discussions and other techniques to ensure that everybody can speak freely. • The Ex Ante methods including problem tree and common vision development were used [10]. A problem tree is a common exercise in which the participants express their perceptions of problems in the sector, and these are linked together, so that it becomes visible how different problems are connected, and in some cases lead to other problems. • Planning, executing and minutes taking was part of facilitation • Meeting minutes transcribed and organised by nonauthor colleague (PhD student) • Perceived outcome: Orchestration of open dialogues
Targeted meeting material	Cattle & pig	• Agenda and meeting material for LL core group and extended meetings prepared by university team, in collaboration with LL participants, relevant for the topic of the meeting. • Material to inspire thoughts, not requiring too much time to read, was shared before the meeting. • Creation of a constructive environment for dialogues across stakeholders with different interests.
Extended meetings across LLs and sectors	Cattle & pig LL and visitors	• Expert meetings: The LLs questioned the connections between AMR as global threat, and AMU in Danish herds, and therefore we set up a seminar with three microbiologists with expertise in AMR (meeting 1) and one with one expert in environmental threats of AMR (meeting 2). • Cross-LL dialogue café to understand systemic factors influencing both factors. Every LL participant invited one colleague or stakeholder who could contribute to and benefit from the dialogues. • Perceived outcome: Establishing a common platform for understanding AMR.
Initiating and testing “tools”	Thematic groups & individual LL partici-pants	• The ‘lab-part’ of the Living Labs consisted of testing initiatives in practice to enhance AMU. The process and results were shared in the LL core group. • Two thematic groups in the pig LL and three in the cattle LL were formed (See Tables 1 and 3) • Individual LL members took initiatives towards prudent AMU which fitted into their job and stakeholder interests (see Table 3). • Perceived outcome: Ownership and sharing of the common vision and aim of the LLs.
Dialogues with stakeholders outside the LLs	Cattle and pig LLs	• Both LLs participated in the annual national sector conferences (see Table 3). • The cattle LL held a calf workshop with sector stakeholders representing slaughter calf producers, abattoirs, advisory services and organisations, to open dialogues on factors influencing AMU in calves. • Perceived outcome: Reaching out to wider stakeholder communities to bring discussions on AMU further into organisations at political and organisational levels.
Round table	Pig LL	• A thematic group in the pig LL took initiative to bring in a broader group of stakeholders to discuss AMU in pig production, discussing four focus areas (‘AMR’, ‘Citizens and consumers’, ‘Alternatives to AMU’ and ‘Breeding for robust pigs’). • The Pig LL core group and 13 invited participants entered into debates, using “Samoan Circle meeting methodology” [14]. The meeting was concluded with common explorations of articulated agreements and disagreements among stakeholders on the debated topics. • A written report summarizing the outcome of the meeting was published [15]. • Perceived outcome: Impact on the further dialogue on AMU among stakeholders. The results were taken to the political level by the think tank ‘Frej’ during winter and spring 2024 [40].
Addressing policies	Cattle LL	• The cattle LL went through a process to address AMU especially in calves, through improved living conditions (e.g. more space per calf) and health promotion focus. The entire LL participated in developing common recommendations forwarded to the policy negotiations planned to take place shortly after. • Perceived outcome: Common insight into how current systemic structures have great impact on the sector’s ability to change policies and production conditions.

Plenary discussions in all meetings (LL core groups, thematic groups and extended event meetings) were voice-recorded and transcribed into detailed meeting notes, which were shared with and confirmed by the LL participants via email and orally in the subsequent meetings. All the meetings were evaluated by allowing the participants to express orally or anonymously in writing how they had perceived the meeting.

Meeting notes were analysed both inductively and deductively, allowing unexpected themes based on spontaneous discussions to emerge. In the present study, one main case was dairy cattle LL and the other was pig LLs (see Table 1).

## Results

### Setting goals and initiating dialogues between stakeholders in the Living Labs

The purpose of the first meeting in the two LL core groups was to set the scene, agree on a common vision for the LL and direct focus towards more prudent use of AMU in the two sectors. Both LLs went through participatory exercises to identify the focus of and visions for their work, among others, by drawing a problem tree [34] (explained in Table 2).

Participants directly related to farming (farmers, vets and farm-advisers) in the pig LL expressed frustration over the amount of public criticism directed at the pig sector [35–37]. They quickly agreed that the Danish pig sector was already managing AMU very well and that the key was to help consumers and citizens recognise this. They suggested that a relevant Danish contribution to the ROADMAP project could be to share with international partners how the Danish pig sector had successfully brought AMU under control and to ensure that current efforts and practices were effectively communicated to Danish consumers and wider society. In Table 1, the main focus area of the pig LL is briefly described. In the cattle LL, the participants expressed that the sector was proactive in continued efforts towards prudent AMU, especially in relation to dairy cows. Their common vision for working in the LL was to support dialogues between different stakeholders, among other things, by developing different dialogue tools to use in practice (see Table 1).

Both LLs formed thematic subgroups, which, in different ways, worked towards the common aim of the LL. One of the aims agreed upon in the cattle LL was to develop practice tools. Table 3 shows examples of these parallel initiatives, which were developed by either thematic subgroups or individual LL participants. During the following years, these groups, and in some cases individual LL participants, took initiatives, which were relevant for them in their everyday practice and position as stakeholders. These different initiatives are briefly described in Table 3.

**Table 3:** Different types of dialogue tools developed by LL participants

Tool developed by individual LL participants or thematic subgroups	The intended purpose of the tool with regard to open dialogues
Initiating dialogues with agricultural colleagues to investigate need for education material on AMU (Cattle LL)	To facilitate and initiate dialogues on AMU among young future farmers, in the first instance through establishing contact with teachers in agricultural colleges.
Establishing pilot learning groups for migrant farm employees (Cattle LL)	To convey knowledge on health, disease and AMU among farm employees in dairy farms.
Making small videos with good examples of reducing AMU (Cattle LL)	To open for dialogues among farmers through demonstration of possible ways forward to reduce the need for antibiotics.
Developing a digital tool to monitor, visualize and benchmark the use of AMU in pig herds (Pig LL)	To improve dialogue between herd managers and employees in a pig herd by providing a digital overview and common platform of information for taking decisions on AMU.
Joint advisory visits between two different types of advisors to improve calf health (Cattle LL)	To improve dialogue in advisory service, addressing different sides of the problems in the herd, and develop solutions which took several factors into account, hence reducing AMU.
Flyers and material to showcase at a stand at the yearly Danish Cattle Conference (Cattle LL) and Pig Conference (Pig LL)	To initiate dialogues about AMU with farmers, advisors, processors and other stakeholders, who participated in the conferences.

These different initiatives are related to the common aim of the first meeting and were discussed at the LL core group meetings, including their perceptions of their outcomes. When analysing the meeting notes, it became apparent that these diverse initiatives were important for the following reasons: (1) they enabled many LL participants to act in ways that were meaningful and valuable for them in relation to their current position, and (2) they involved the organisation behind the LL participant, and in doing so they created connections between their stakeholder organisation and the work of the LL.

### Participant feedback on the meetings in the LL core groups

When these meetings were evaluated in the LL core groups, the participants agreed that the participatory processes helped them understand the complexity of AMU and that discussions in the LL core groups provided a holistic view of systems and interests affecting the AMU. Many of the stakeholders knew each other and had different types of collaboration over time, but according to their feedback, they had not participated in a guided discussion with the aim of AMU reduction, which they generally welcomed as useful and relevant. Some issues around AMU were sensitive because of different interests and perceptions of e.g. what was ‘prudent use’. There were sometimes unspoken agreements about AMU, e.g. the necessity to use it in certain situations. Table 2 shows the perceived outcomes of the different methods used by the LLs to achieve their goals on the basis of participant feedback.

### Dialogues across LLs and sectors

Dialogues were also initiated between the two sectors through three workshops, which involved participation from both LL core groups, as explained in Table 2. The first two workshops were set up primarily on requests from the pig LL, but with the possibility of participation from both LLs. The request was made because the participants disagreed on the connection between AMU in Danish farms and the global threat of AMR to public health. In these workshops, a total of four scientists with expertise in AMR gave talks on AMU in farm animals from a one-health perspective, and they participated in discussions on potential risks of AMU in farm animals for human and environmental health. The third workshop was shaped as a ‘dialogue café’ and was set up on request from both LL core groups with the aim to facilitate exchange of experiences and encourage discussions across the two sectors. Additional guests representing different stakeholder organisations were invited. In total, 33 participants from different stakeholder organisations (16 related to the cattle sector and 17 related to the pig sector) joined the event, where they discussed five different themes in smaller groups.

Stakeholders from both sectors agreed on a definition of prudent AMU as “using as little as possible, but as much as necessary”. During the meeting, it became clear that the participants did not agree on how much would be necessary or how little would be possible. Based on the meeting notes taken at discussion tables during this meeting, the discussions continued in the two LLs.

From these meeting notes, it became clear that stakeholders from the two sectors perceived their possibilities for change differently, and that they realized that the perception of prudent AMU differed between the two sectors. Stakeholders from the pig sector are under pressure from strict regulations and negative attention from society, e.g., in relation to issues such as methicillin-resistant *Staphylococcus aureus* (MRSA). Furthermore, they felt that strategies to reduce AMU beyond what was already achieved would make the production of pork more expensive and therefore unrealistic in a global market situation.

The participants in the cattle LL emphasised that the dairy sector has continuously decreased its AMU over recent years, particularly in dairy cows. However, AMU in calves for slaughter was identified as critical for the cattle sector. The LL participants talked about how the living conditions and AMU in calves from dairy herds had been debated over the last decade, especially in relation to early killing of male calves. The LLs discussed how calves not intended for milk production had so little economic value that it was not economically viable to let them stay in the dairy herd for more than a few weeks. Therefore, these calves were transported to calf fattening farms at a young age, where they were mixed with calves from other herds. This increased the risk for infections and thereby high AMU. The LL participants agreed that this was something they needed to work on in the LL, and as it was a systemic problem in the sector, it needed to be addressed on a structural level.

This debate across the two sectors led to a shared realisation among the participants in the cattle and the pig LLs that there were some commonalities between the pig and the calf sectors as both were under economic pressure.

### Two multistakeholder dialogues as outcomes of LL work

#### Articulating agreements and disagreements on future AMU policies in the pig sector

A thematic group in the pig LL took the initiative to bring in a wider group of stakeholders to discuss AMU in pig production. They identified 4 focus areas (‘AMR’, ‘Citizens and consumers’, ‘Alternatives to AMU’ and ‘Breeding for robust pigs’) and invited 13 external participants for this event. The participants were all experts in at least one of the four focus areas. The meeting design was inspired by the dialogue method known as the “Samoan Circle meeting process” [38], with four speaker chairs that participants could queue for. Each of the debates was introduced with a two-minute talk on the specific theme from the invited experts, followed by a debate. While the severity of AMR was emphasised, many participants were still sceptical of the connection between Danish pig production and the global risk of AMR. Stakeholders, who otherwise disagreed on some of these matters, seemed to find areas of agreement, e.g., industrial breeding experts agreed that increasing litter sizes could be counterproductive to lowering AMU.

A written report summarising the outcome of the meeting in terms of agreements and disagreements and the arguments behind them was confirmed by participants and published [39]. This report inspired the final debate meeting for a wider selection of stakeholders across the two sectors. Furthermore, the think-tank Frej used the report as inspiration when organizing a public event to debate AMR and farming at a political level [40].

### Formulating recommendations for future AMU policies in the dairy and calf sectors

The focus on calves became stronger during the work in the LL, and it became increasingly clear for the dairy cattle LL participants that the way in which the calf industry was organized contained systemic barriers for reducing AMU. The inequity between future replacement heifers and calves, which were intended as slaughter calves, was also reflected in the way in which the highest-quality colostrum was typically fed to female calves. The dairy cattle LL invited a larger group of stakeholders to a workshop regarding calves from dairy herds, where issues and possibilities were discussed, and they followed up with a special focus on calves when they decided to formulate policy recommendations for an upcoming political agreement (Food and Veterinary agreement 2024–2027 [41], which would define the guidelines for antibiotic use in cattle. The thematic subgroup developed a draft over half a year, after which it was debated in the LL core group for agreement before being submitted to the relevant authorities. One LL participant, representing a farmer-owned veterinary consultation company, emphasized that the policy recommendations should not “pull the carpet from under the farmers, who were already under economic pressure”, as directly quoted by this LL-participant. The participant specifically referenced the recommendation of a space requirement of 3 m2 per calf in the housing system (Table 4), which was too demanding. Other LL participants argued strongly for a recommendation that each calf should have more space, referring to recent research and reports that pointed to the connection between space and staying free of disease. After negotiations and exchanges of different arguments, the LL participants agreed upon a final version of the legislation recommendations. Table 4 shows the headlines of the final version.

**Table 4:** Nine recommendations elaborated by the dairy cattle LL and submitted to the Danish Veterinary and Food Administration, May 2023

Issue raised	Recommendations
Regarding medication and AMU:	• Establishment of a yellow card initiative targeting calves *^)^. • Only use narrow-spectrum antibiotics for cows. • Oral medication to calves should not be allowed, as a starting point. • Efforts should target the herds with the highest AMU • Allowed levels of AMU should be different between calves in dairy herds and calves in slaughter calf herds
Regarding preventing disease and supporting the health of cows and calves:	Specific space requirements (min. 3m2/calf) should be applied by legislation Measuring the immunity of calves should be part of the standard herd advisory consultancy Digital registration of AMU should be mandatory to ensure professional and homogenised data analyses to follow up on how antibiotics are used. Knowledge about AMR and AMU should be disseminated at agricultural colleges

*^)^ Since 2009, the yellow card initiative targeting pig farmers with a high AMU has been in place. The initiative sets national threshold limits for the use of antimicrobials in each age group (https://foedevarestyrelsen.dk/dyr/dyresundhed/laegemidler-til-dyr/anvendelse-af-laegemidler-til-dyr/antibiotika/gult-kort-ordningen)

This process demonstrated how different interests and perspectives on time frames for perceived solutions from the stakeholder organisations could influence negotiations between LL participants when making concrete recommendations.

### Common reflection on how Living Labs worked as rooms of negotiation towards Lowering AMU

The LL processes made explicit how the participants had several roles, because they represented their organisations at the LLs and represented the LLs at their organisations. At the same time, they acted as individuals with personal experiences and interests in the topic. Participants had to balance being an individual and being a representative throughout the LL process. At times, participants stated that they were unsure whether their organisations agreed with them on certain issues, but this was the way they saw it, e.g., when talking about visions for the future or solutions to AMU that would require less transport of animals. On other occasions, participants explicitly referred to standpoints expressed by their organisation or stated that they could not go further without confirmation from their organisation or institution.

## Discussion

### Perceptions of AMR risks in relation to Danish animal farming

Over centuries, Denmark has developed large pig and dairy sectors for export of pork and butter, with high efficiency and production as main focus areas [42, 43]. Over the last three decades, Denmark has strongly focused on ensuring well-documented AMU and has often been portrayed as a country with low AMU, compared to many other countries with similar highly productive and industrial animal farming [44]. This low level has, among other things, been explained by a strong focus in the Danish legislation and regulation on control over and registration of AMU [44], although other sources question this and indicate that imprecise registrations and at times underreporting have also occurred in Denmark [45].

As mentioned above, the LL members agreed on the statement “as little as possible, but as much as necessary” and some claim that this was already included in their practice. This statement has been widely referred to in the sector for years [46], and perhaps it indicates that what they already do is sufficient. In different contexts, this has often been shown to be a general perception: it is sufficient to “do as they always did” [47]. With regard to AMU, it is documented in several contexts that decisions to initiate antibiotic treatments are highly influenced by different perceptions, attitudes and approaches to disease [48–52].

### Recognising the multiple pressures and the systemic nature of antimicrobial use

Throughout the process, the LL participants gradually acknowledged the importance of other stakeholders than farmers and veterinarians, which helped them focus on possible innovations and solutions beyond the farm level, e.g., legislation, market situations, different actors’ responsibilities and interactions between human and animal medicine. Some of the systemic challenges were related to international trade and export markets, e.g., of live animals, meat and dairy products. The discussions and events in the LLs confirmed that identifying solutions to systemic challenges, such as AMU, required time and effort for the stakeholders to negotiate and find common ground to try to influence entire systems.

### The threat of AMR and the One-Health perspective on AMU

As stated in the introduction, a major initial argument for this project was the growing threat of AMR, including the One Health perspectives related to this threat. At the end of the project, the connection between AMR at a global level and Danish dairy and pig farming was still questioned. Much debate on responsible or prudent AMU has focused on the farm and veterinary levels [e.g., 48, 53], as they are perceived as the end-users of antibiotics. Studies have shown that farmers do not see a connection between the threat of AMR and on-farm AMU in neither pigs [54] nor cattle farming [55, 56]. In the two Danish LLs, much focus was also on the farm level, but by taking broader views and involving different stakeholders and experts, more complex perspectives were discussed and added to a common understanding of the One Health perspectives of AMU and AMR.

### Opening dialogues and negotiating rather than innovating, or innovation through withdrawal?

Both pig production and calves raised for meat share challenges of high economic pressure, which restricts their ability to implement costly (in time or money) solutions or make major systemic changes. In the calf sector, systemic challenges have been debated, but no solutions have been identified. A common realisation that antimicrobials cannot be taken away or reduced significantly without profound systemic changes has been reported in other studies [57, 58]. Significantly reducing AMU means taking something away on which the sectors have relied for decades to mitigate the negative effects of infectious diseases and increased production efficiency. While much of the literature on LLs focuses on innovations, at an early stage of the development of the LL concept, different types of innovations could be relevant in different contexts. However, the theory of ‘innovation through withdrawal’ [59] states that innovations are forced when elements that are important for maintaining a certain system are taken away. This describes the situation regarding AMU well: efficient large-scale pig and calf production in Denmark is partly shaped by the possibility of providing antibiotics. Antibiotics are permitted only for the treatment of diagnosed diseases and not to prevent outbreaks of infectious diseases. Nevertheless, the risk of disease connected to practices such as mixing young animals from different herds is well recognised, and yet, this practice is what keeps the calf industry possible. It was clear in this project that systemic changes were potentially painful and that enforcing them would have enormous consequences for some stakeholders. Such systemic changes will both require innovations (e.g., new practices, new education initiatives, new markets) and negotiations (e.g., legislation and price levels). This indeed involves international governance systems and the entire value chain and extends far beyond simple innovations, which requires a multistakeholder approach far beyond the scope of the LLs in this study.

### Is the method of Living Labs valuable in fostering AMU changes in animal husbandry?

As described, issues around AMU were sensitive topics with potential conflicts and unspoken practices and agreements. This study highlights the importance of bringing a range of different stakeholders together to initiate new dialogues and identify common visions and understandings of the challenges and problems of different groups of actors. The LL approach offered through its participatory multistakeholder methodologies the possibility to foster such dialogues, which have the potential to lead to a common recognition of the complexity and magnitude of AMU in animal farming. This study used LLs as a participatory action research (PAR) approach to focus on the inclusivity and involvement of stakeholders who are potentially affected by such actions. This ensures that LL participants can influence decisions that might impact them [60]. PAR can potentially make academic research more applicable to real-world issues by integrating academic and real-world knowledge and experiences [31]. When establishing the LLs in this project and when selecting the participants, we focused on these elements. Furthermore, we acknowledge that the time frame of the research project and work with the LLs was limited to approximately 30 months.

In this study, the LL activities and dialogues sparked conversations and collaboration across sectors, which provided new insights. Stakeholders understood how the pig and calf industries share similarities that they had not articulated before, e.g., that high numbers of both calves and pigs were mixed into new groups as a consequence of the industry structure. This often led to a high AMU to prevent or solve problems with infections such as diarrhoea or pneumonia. In some cases, the LL coordinators had to address dependencies and power imbalances between the participating stakeholders’ business interests. The ability to articulate differing interests and disagreements between stakeholders appeared promising for fostering change in a sensitive area. The LL processes emphasise the relevance of the point at which LLs are ‘strongly place-based, which enables them to work with the most relevant solutions in each situation’ [61].

Importantly, the LLs in this project were not initiators or drivers of AMU reductions in Denmark but rather worked in synergy with many existing initiatives, which also involved the LLs in a high-lighted “sphere of influence” [22]. In this way, the meetings, initiatives and events organised by the LLs served as a platform for continued dialogue and positive collaboration to reduce AMU. There were some positive developments for reducing AMU between 2020 and 2023, and some of the actors in the LLs were directly involved in initiating projects and sector decisions, leading to continued AMU reductions, although we cannot establish a causal relationship between LL activities and these developments.

### Critical preconditions of using LLs: time and facilitation

Reaching a common understanding– e.g., of the systemic nature of AMU– was time-consuming. Trust between participants in processes of mutual learning and knowledge exchange is crucial [26, 62, 63]. The small group size in this study proved to be advantageous many times. For example, there were several sensitive viewpoints among the different stakeholders when discussing AMU reductions, and some stakeholders had business interests or highlighted financial concerns because many solutions to AMU reduction are more expensive. Veterinarians valued the ability to write prescriptions without restrictions and explained how they feared the loss of autonomy if more restrictions were put in place. The fact that different stakeholders could openly raise these viewpoints and concerns could indicate a high level of mutual trust. The two LLs set out to foster changes towards a more prudent AMU through genuine dialogues and social innovations between stakeholders. During the process, the participants articulated clearly how the agricultural sector is highly influenced by markets, price settings of products and labour, and politically determined regulations. To different degrees, the participants in both LLs expressed how they felt that they had little control over these factors, e.g., through international markets, leading to long transports of pigs and calves. In that light, it seemed ‘easier’ to discuss AMU at farm or veterinary practice levels rather than at the systemic level. Moving the focus from farm and veterinary levels to a systemic and value chain level also pointed to the relevance of inviting other stakeholders into the LL, who had influence, e.g., at governance level. The initiative taken by the cattle LL to provide recommendations at policy level illustrates that they felt it was worthwhile to invest time and effort to influence the process as an LL. However, it may also suggest that it could be relevant and fruitful to have representatives from the policy level as part of the LL. Recent studies at the systemic level have revealed the influence of many other stakeholders and actors in the animal farming sector and industry on AMU [57, 61]. Perhaps even international LLs would be needed to open dialogues and foster changes at this level, as calves and pigs from Denmark are transported to many other countries to be fattened for slaughter. Finding common solutions to those challenges may require a much wider LL to open dialogues across borders and gradually innovate solutions for different farming contexts. In other projects working with LLs, it was also useful to engage with actors who did not immediately identify themselves as direct stakeholders in relation to the sector or topic in focus. For example, a study on organic vegetable markets in a Norwegian region has been conducted, where it was a great advantage for the project to engage with ‘outside actors’ to create a common understanding of the complexity of the organic vegetable system, because the stakeholders ‘from outside’ contributed to creating a fuller understanding by adding new and different views on the topic [64].

It was also clear that the interactions between the stakeholder organisations and their participation in an LL with this focus developed over time. This suggested that a newly established institution such as a “Living Lab” needs to work for some time to be recognised in the wider sectoral landscape and to attract the attention of influential stakeholders. The Living Lab only works if the involved stakeholders can contribute with their active involvement in addressing issues such as AMR and prudent AMU. These are issues, which have enormous long-term consequences but in the current situation, seem less urgent than, e.g., economic pressure in a sector.

As described above, the facilitation of the LL processes was carefully planned to allow dialogues and confidentiality (see Table 2). Facilitation has been described as essential to the functioning of the LLs, to maintain the exchange and negotiate evolving contextual factors [63], and to create the conditions for dialogues and processes leading to efficient and meaningful change [65].

### Limitations of the study

This project existed within the limited time frame of an EU project and was initiated during the COVID-19 pandemic, which put some restrictions on activities and developments. The too short funding cycles have previously been pointed to as a limitation for research projects [66], not allowing time for meaningful change. When allowing a sufficient time for processes and development of common understandings, a LL approach could include a wider range of stakeholders, e.g. authorities and governance related stakeholders, more organisations (e.g. citizen groups, consumer organisations, or animal protection organisations), and stakeholders with wider perspectives on environmental and social issues. As discussed above, this would have been very relevant in relation to overcoming systemic barriers to reducing AMU.

This project was a participatory action research study, which means that participants partly determined the course of the developments. This can be seen as a strength, but also a limitation of the study. It is a strength, because new perspectives, which are relevant for the participants, are brought into and enrich the research. The relative unpredictability of how the project develops can be seen as a weakness or a limitation, because it might bring some inconsistencies and changes into the process that was originally planned.

## Conclusions

The LL multistakeholder approach fostered important conversations and insights between central stakeholders in the Danish dairy cattle and pig sectors, where the complexity of AMU in both pig and cattle sectors became apparent and articulated. Several barriers toward prudent AMU were identified to be on a systemic level. This included governance and market conditions, and thereby dependence on international cooperation to enforce systemic changes in the sectors. The need to negotiate potential solutions lies in facilitating dialogue processes in environments of confidence and trust and with the involvement of a range of committed and influential stakeholders. An LL structure that consisted of a limited number of committed participants representing different stakeholder organisations provided this atmosphere. Differences and similarities between the two sectors became visible, which pointed to the relevance of collaboration across sectors. Initiatives opened for technical and social innovations, although the time frame of this study did not allow for long-term cycles of development.

## Electronic supplementary material

Below is the link to the electronic supplementary material.

Supplementary Material 1

## Data Availability

The datasets, including meeting notes, transcripts and reports used and/or analysed during the current study, are available from the corresponding author upon reasonable request.

## Declarations

### Authors’ Information

MV (DVM, MSA, PhD) is a senior researcher with focus on dairy farming, anthropology and qualitative research. MS (MSc in agriculture, PhD) is a special consultant at the International Centre for Research in Organic Food systems. HK (DVM, PhD) is associate professor with focus on pig farming and mixed methods. MAK (DVM, PhD) is associate professor in epidemiology, herd health management and animal health economics.
