# Supplementary material for: Living labs opened dialogues about antibiotic use in dairy cattle and pig sectors - Insights from a Danish case study based on participatory action research
Source: Acta Vet Scand. 2025 Jun 17;67:33. doi: 10.1186/s13028-025-00816-7 (PMC12172369; doi:10.1186/s13028-025-00816-7)
Supplement: Supplementary file 1 — Supplementary Material 1 [file 13028_2025_816_MOESM1_ESM.docx]

**Supplementary materials**

Interview Guide: Key Person Interview (example cattle)

(translated from Danish to English using AI-tool Copilot)

Before the interview: Participants have received a document with information about the project and a form for checking acceptance.

Introduction

ROADMAP (Rethinking Of Antimicrobial Decision-systems in the Management of Animal Production) is about understanding how different actors, regulations, and traditions affect health management and antibiotic use in agriculture. We will look at how decision systems around health management and antibiotic use function.

The main purpose of the interview is to describe and map how antibiotic use in livestock in Denmark is influenced by different actors (such as herd veterinarians, legislators, and supply chains). This will result in a “Stakeholder Map” that describes the roles, degree of influence, and mutual impact of the actors.

Key person interviews will also be used as background information and idea generation in connection with the establishment of Living Labs.

**Interview**

Yourself and Your Role(s)

How do you see your own role in relation to the use of antibiotics in dairy herds, both in specific cases and more generally?

How has it developed over time from your perspective – both your role and the discussion itself?

How do you see your own role in relation to organic versus conventional herds, both in terms of health promotion and disease management?

What tools in your daily life do you find most useful in achieving minimal antibiotic use in dairy herds?

Stakeholders involved in your decision making

Actors with General Influence a) Who do you specifically collaborate with regarding decisions about antibiotic use in dairy herds / the dairy sector? b) If we move away from your role and immediate collaborators, who else do you immediately think of as ‘key persons’ influencing decisions about antibiotic use in dairy herds (organic vs. conventional)? c) … and in the entire sector? For each actor mentioned under a), b), and c):

How do they influence? How do you experience their impact on the discussion/decisions? How do you see your own communication/interaction with them (any synergies, conflicts, etc.)? How do you feel influenced by them?

Include Stakeholder Map Specific actors with influence on antibiotic use: If the following actors are not mentioned by the interviewee, ask about the following:

What about economic conditions/bank influence?

How do you view SEGES in this context? How have you experienced SEGES’s role over time?

Antibiotic use in Denmark

How do you think our way of selling and distributing antibiotics in Denmark affects decisions to use antibiotics and overall consumption? How do you see the historical development (1995 / 2010 … if you know the development that far back)?

How do you view the role of the Danish Veterinary and Food Administration in ensuring low antibiotic use in livestock?

What experiences do you have with whether benchmarking or other tools can influence decisions and strategies in herds?

What about calf breeders/buyers – do you have experiences with them, and how do you see their situation and role in minimizing antibiotic use?

Inspiration from Outside and Future Prospects

How do you see the future development of the dairy sector? How will this development affect antibiotic use?

How do you personally want the development to proceed? What visions and wishes do you have for future development in terms of health promotion and disease management in dairy farming?

How do these visions affect your daily work and your motivation for your daily work?

Can you describe what you consider to be ‘responsible’ vs. ‘irresponsible’ use of antibiotics? Please provide examples. How do you perceive others define ‘responsible’ vs. ‘irresponsible’ use of antibiotics? How do you view your opportunities to … influence the understanding of ‘responsible/irresponsible’ use of antibiotics? … and change current practices if you believe they should be changed in relation to responsible/irresponsible use at present? Where do you find inspiration for the future of dairy farming? (organic, conventional, special productions, others …)

Concluding: what do you think is important within this topic that we did NOT manage to touch upon during this interview?
